# Family History of Hypertension and the Risk of Overweight in Japanese Children: Results From the Toyama Birth Cohort Study

**DOI:** 10.2188/jea.JE20130149

**Published:** 2014-07-05

**Authors:** Jufen Liu, Michikazu Sekine, Takashi Tatsuse, Shimako Hamanishi, Yuko Fujimura, Xiaoying Zheng

**Affiliations:** 1 Institute of Reproductive and Child Health; Ministry of Health Key Laboratory of Reproductive Health; Department of Epidemiology and Biostatistics, School of Public Health; Peking University, Beijing, China; 1 北京大学公衆衛生大学院疫学生物統計学講座; 2 Department of Epidemiology and Health Policy, Graduate School of Medicine and Pharmaceutical Sciences, University of Toyama, Toyama, Japan; 2 富山大学大学院医学薬学研究部疫学健康政策学講座; 3 Institute of Population Research; Peking University, Beijing, China; 3 北京大学人口学研究所

**Keywords:** family history, hypertension, overweight, the Toyama study

## Abstract

**Background:**

Family history can be a useful screening tool in the assessment and management of the risk for noncommunicable disease. However, no data have yet been reported on family history of hypertension and its effect on children’s overweight.

**Methods:**

A total of 7249 Japanese children enrolled in the Toyama Birth Cohort Study were followed until 2002 (mean age: 12.3 years). Family history of hypertension was ascertained by asking children’s parents whether children’s biological parents or grandparents had doctor-diagnosed hypertension. Child overweight was defined according to international criteria for age- and sex-specific body mass index.

**Results:**

The prevalence of child overweight at age 12 was 21.7% for males and 15.9% for females. After adjusting for family structure, parental employment status, and lifestyle factors, we found that a maternal family history of hypertension was positively associated with the risk of child overweight at age 12 (adjusted odds ratio [OR] 1.21, 95% confidence interval [CI] 1.04–1.39). The adjusted OR increased from 1.16 (95% CI 0.99–1.35) to 1.42 (95% CI 1.04–1.92) to 4.75 (95% CI 1.35–16.69) as the number of family members with hypertension increased from 1 to 2 to 3, respectively. There was no significant difference in the prevalence of overweight between children with a paternal family history of hypertension and those without.

**Conclusions:**

A maternal family history of hypertension was positively associated with the risk of overweight in children at age 12.

## Abstract (ja)

**【背景】:**

家族歴は、生活習慣病のリスク評価や管理に有用な情報である。しかし、高血圧の家族歴の子どもの過体重への影響については、まだ報告がない。

**【方法】:**

富山出生コホート研究の対象者のうち、2002年の調査まで追跡できた7249人（平均12.3歳）を対象とした。高血圧の家族歴に関する情報は、子どもの両親や祖父母に高血圧と診断された人がいるかどうかを、子どもの保護者から得た。子どもの過体重の有無は、年齢性別ごとの体格指数を用いた国際分類方法で判断した。

**【結果】:**

12歳の時の過体重の有病率は男子で21.7％、女子で15.7％であった。家族構成、両親の雇用状況、生活習慣要因を調整した後、母方に高血圧の既往がある場合の12歳の時の子どもの過体重に対するオッズ比は1.21（95％信頼区間：1.04‐1.39）であった。母方の高血圧者の数の12歳の時の子どもの過体重に対するオッズ比は、高血圧者が１人の場合で1.16（0.99‐1.35）、高血圧者が２人の場合で1.42（1.04‐1.92）、高血圧者が3人の場合で4.75（1.35‐16.69）であり、母方の高血圧者の数が増えるほど子どもの過体重に対するオッズ比は上昇した。父方の高血圧の家族歴と子どもの過体重との関係は有意ではなかった。

**【結論】:**

母方の高血圧の家族歴は、12歳の時における子どもの過体重のリスクとなる。

## INTRODUCTION

Given the growing prevalence of high-fat and high-calorie diets and the lack of adequate physical exercise, the worldwide burden of noncommunicable disease is increasing. Hypertension, a common noncommunicable disease, is one of the most common risk factors for cardiovascular disease. Hypertension no longer affects just older adults; the prevalence and rate of diagnoses of hypertension in children and adolescents is increasing, in part because of the increasing prevalence of childhood obesity as well as growing awareness of this disease.^1^ There have been dramatic increases over the past several decades in the prevalence of childhood obesity in Japan and other industrialized nations. A longitudinal study from the Japan Public Health Center showed that an increasing prevalence of obesity was observed among younger generations of men.^2^ Preventing obesity in children would help reduce the prevalence of hypertension, diabetes mellitus and cardiovascular disease in the near future.

Family history is a consistent and independent risk factor for many common chronic diseases, and professional guidelines usually include the use of family history to assess health risk, initiate interventions, and motivate behavioral changes.^3^ Hypertension and obesity share several physiopathologic abnormalities that are related to renal handling of sodium.^4^ Although family history of obesity, rather than family history of hypertension, may be more closely associated with obesity in children, the differences in disease sensitivity between children and adults may lead to different manifestations of the disease. Children may be more physiologically sensitive to obesity (eg adipocyte proliferation) and more resistant to hypertension (eg flexibility of vascular wall). For their mothers, leanness, rather than obesity, might be more common, and the association between the risk factors for hypertension and presence of obesity in mothers may be weak. Similarly, in the generation of their grandparents, hypertension might also be more common than obesity.

Although weight and blood pressure have been found to be associated, the correlation coefficient is approximately 0.4 in young adults but is smaller in older adults. It is likely that weight interacts with various factors controlling blood pressure at different points over a lifetime.^5^ These possible differences in disease sensitivity across generations and the fact that correlation between weight and blood pressure vary by age suggest that family history of hypertension may be a useful proxy for detecting the risk of obesity in children.

A study of Gambian adults found that subjects with a family history of hypertension had a higher body mass index (BMI) and an increased risk of obesity than those without a family history of hypertension.^6^ A study from India found that BMI was significantly higher in participants with a family history of diabetes than in those without such a family history, but BMI was not significantly higher in participants with a family history of hypertension.^7^ However, both of these studies focused on young adults (aged 22–35 years), and the sample size in the second study was relatively small (*n* = 67), so further research on the association between family history of hypertension and obesity is needed, especially in children.

The aim of the present study was to evaluate the association between a family history of hypertension and overweight in children in a Japanese population. We hypothesized that a family history of hypertension increases the risk of child overweight and that there might be interaction of family history of hypertension and parental overweight with child overweight.

## METHODS

### Subjects

Subjects were from the Toyama Birth Cohort Study, a prospective, longitudinal survey of 10 438 children born between April 2, 1989, and April 1, 1990, in Toyama Prefecture, Japan.^8^ This birth cohort was followed up in 1992 (Phase 1, age 3), 1996 (Phase 2, age 6), 1999 (Phase 3, age 9) and 2002 (Phase 4, age 12).

Questions about family history of hypertension were asked in Phase 1, including questions about the mother and father and the grandmothers and grandfathers from both sides. Information on children’s weight and height, as well as parental body measurements, was collected at every phase of the study.

In Phase 1, an invitation letter and a questionnaire were sent to the subjects through local public health centers. In Phases 2–4, an invitation letter and a questionnaire were sent to the subjects through their schools. Participants and their parents answered the questions and returned them in a sealed envelope. Written informed consent was obtained from the parents of the participants. All participants and their parents participated voluntarily. The Toyama Birth Cohort Study was approved by the Ethical Committee of the Toyama Medical Pharmaceutical University.

### Sociodemographic characteristics and lifestyle factors

Family structure (living with two parents, parents and grandparent[s], single parent) and parents’ employment statuses were collected in Phase 1, while lifestyle factors were collected in Phase 4. Children’s age, sex, weight, and height were collected in Phase 1 and Phase 4.

The heights and weights of the children at age 3 were based on anthropometric measurements that were undertaken at regional public health centers by trained public health nurses according to the protocol of the Law for the Health of Mothers and Children.^8^ The heights and weights of the children at Phase 4 were taken from records of a physical examination taken within the past 4 months, in which height was specified to the nearest 0.1 cm and weight to the nearest 0.1 kg. A previous study revealed that height and weight values as reported by parents were close to actual measurements (correlation coefficients ranged from 0.90 to 0.96 for height, 0.95 to 0.99 for weight, and 0.86 to 0.97 for BMI).^9^ Parental heights and weights were self-reported in questionnaires. BMI was used as an index of overweight in this study and was calculated as weight (kg)/height^2^ (m^2^). Parental overweight was defined as a BMI > 25 kg/m^2^ according to criteria from the World Health Organization.^10^ Child overweight was defined according to international criteria on age- and sex-specific BMI cutoff points based on an adult BMI > 25 kg/m^2^.^11^ These values were 17.9 kg/m^2^ for males and 17.6 kg/m^2^ for females at age 3 and 21.2 kg/m^2^ for males and 21.7 kg/m^2^ for females at age 12.

We used the frequency of eating breakfast as a surrogate of the eating patterns of daily life; this variable was measured on a 4-point scale (very often, often, sometimes, seldom). For physical activity, we used the frequency of physical activity (very often, often, occasionally, never). Average amount of sleep per day was also measured (<7 hours, 7–8 hours, 8–9 hours, >9 hours/night). The validity of the lifestyle questionnaire was confirmed in a previous study. Subjects with a high frequency of physical activity (those who reported doing physical activity very often) were significantly more likely than those who occasionally or never did physical activity to show an increasing trend in energy expenditure originating from physical activity, mean steps, and mean activity counts per day originating from an Actiwatch (*P* < 0.05 for a linear trend test).^12^ The correlation coefficient between subjective and objective records was 0.97 (*P* < 0.001) for assumed amount of sleep, and the difference between subjective and objective records was 8.19 min (95% CI, 4.93–11.45) for assumed amount of sleep.^13^

### Family history of hypertension

Family history of hypertension was ascertained by asking whether children’s biological parents or grandparents had doctor-diagnosed hypertension in Phase 1. The advantages of family history in studying risk factors for disease over other genomic tools include its lower cost, greater acceptability to subjects and researchers, and reflection of both genetic and environmental factors.^3^ Although the sensitivity and specificity of family history on hypertension by questionnaire compared with accurate methods (eg clinical charts or research database) vary, results from a U.S. study showed that the sensitivity of self-report parental and spousal history of hypertension was 76% and 77%, with specificity was 84% and 96%, respectively.^14^

### Statistical analysis

To evaluate the association between child overweight and family history of hypertension as well as other correlated factors, we performed chi-square analyses. Adjusted odds ratios (ORs) and their corresponding 95% confidence intervals (CIs) for being overweight at age 12 were calculated from logistic regression models after adjusting for potential confounders. The interaction of family history and parental overweight on child overweight was assessed on an additive scale, using the relative excess risk due to interaction, the proportion attributable to the interaction, and the synergy index developed by Andersson.^15^ Statistical analyses were conducted using SPSS version 18.0 (SPSS Inc., Chicago, IL, USA). Cases with missing values for any of the independent variables were excluded in the final logistic regression. A two-sided *P* value of <0.05 was considered statistically significant.

## RESULTS

Descriptive statistics for the subjects in Phase 4 are presented in Table 1. A total of 7249 children were followed from Phase 1 to Phase 4. The children had a mean age of 12.3 (standard deviation [SD] 0.52) years at Phase 4, and their mean birth weight was 3141.4 (SD 420.15) g. Mean BMI at age 12 was 19.5 (SD 4.04) kg/m^2^ for males and 19.2 (SD 3.35) kg/m^2^ for females. A total of 60% of children lived with parents and grandparents, and 93% had sibling(s). Moreover, 98% of fathers and 53% of mothers were employed full time. In addition, 86% of children ate breakfast every day, and more than 60% of children engaged in physical activity often. About 60% of children got more than 7 hours of sleep per night. There were significant differences between males and females in frequency of eating breakfast, frequency of physical activity, and amount of sleep (Table 1).

**Table 1.** Descriptive statistics for the subjects

Characteristic		Total (*n* = 7249)		Males (*n* = 3634)		Female (*n* = 3615)		*P*
		
		*n*	%	*n*	%	*n*	%	
Age (mean ± SD)		12.3 ± 0.52		12.3 ± 0.49		12.2 ± 0.54		0.004
Body mass index (mean ± SD)		19.4 ± 3.71		19.5 ± 4.04		19.2 ± 3.35		0.039
Family structure	Parents and grandparent(s)	4184	57.7	2091	57.5	2093	57.9	0.228
	Two parents	2673	36.9	1340	36.9	1333	36.9	
	Single parent	252	3.5	112	3.1	140	3.9	
	Missing	140	1.9	91	2.5	49	1.4	
Father’s employment	Full time	6651	91.8	3348	92.1	3303	91.4	0.434
	Part time	31	0.4	17	0.5	14	0.4	
	Not employed	61	0.8	26	0.7	35	1.0	
	Missing	506	7.0	243	6.7	263	7.3	
Mother’s employment	Full time	3710	51.2	1844	50.7	1866	51.6	0.424
	Part time	2182	30.1	1108	30.5	1074	29.7	
	Not employed	1023	14.1	498	13.7	525	14.5	
	Missing	334	4.6	184	5.1	150	4.1	
Siblings	Yes	6623	91.4	3301	90.8	3322	91.9	0.957
	No	494	6.8	244	6.7	250	6.9	
	Missing	132	1.8	89	2.4	43	1.2	
Frequency of breakfast	Very often	6306	87.0	3210	88.3	3096	85.6	0.000
	Often	590	8.1	250	6.9	340	9.4	
	Sometimes	205	2.8	91	2.5	114	3.2	
	Seldom	72	1.0	38	1.0	37	1.0	
	Missing	73	1.0	45	1.2	28	0.8	
Frequency of physical activity	Very often	1698	23.4	1096	30.2	602	16.7	0.000
	Often	3120	43.0	1661	45.7	1459	40.4	
	Occasionally	2017	27.8	732	20.1	1285	35.5	
	Never	310	4.3	88	2.4	222	6.1	
	Missing	104	1.4	57	1.6	47	1.3	
Amount of sleep	<7 hours	1363	18.8	547	15.1	816	22.6	0.000
	7–8 hours	3161	43.6	1485	40.9	1676	46.4	
	8–9 hours	2116	29.2	1215	33.4	901	24.9	
	>9 hours	512	7.1	325	8.9	187	5.2	
	Missing	97	1.3	62	1.7	35	1.0	

SD, standard deviation.

The proportions of positive paternal (including father and grandparents on the father’s side) and positive maternal (including mother and grandparents on the mother’s side) family history of hypertension were 35.7% and 34.4%, respectively. These values did not differ statistically between males and females. The prevalence of overweight was 21.7% and 15.9% at age 12 for male and female children, respectively. The prevalence of overweight differed significantly between males and females (*P* < 0.05).

Family history of hypertension was also closely associated with parental obesity. Results showed that the Pearson correlation coefficients between hypertension of grandparents on the maternal side and presence of maternal obesity was 0.024 (*P* = 0.055), the Pearson correlation coefficients between hypertension of grandparents on the paternal side and presence of paternal obesity was 0.046 (*P* = 0.001). The fact that the *P*-value is marginally significant on the maternal side may partly due to the low prevalence of maternal obesity (around 7%). Table 2 shows the characteristics of participants stratified by maternal hypertension history. There was no significant difference in sex or socio-economic and lifestyle factors between participants with maternal family history of hypertension and those without, except that participants with maternal family history of hypertension had higher BMI than those without (*P* < 0.05).

**Table 2.** Characteristics of participants by maternal hypertension history

Characteristic		Maternal hypertension history^a^				*P*

		Yes (*n* = 2475)		No (*n* = 4730)		
	
		*n*	%	*n*	%	
Age (mean ± SD)		12.3 ± 0.50		12.3 ± 0.53		0.675
Body mass index (mean ± SD)		19.5 ± 3.79		19.3 ± 3.67		0.011
Sex	Male	1233	34.1	2384	65.9	0.638
	Female	1242	34.6	2346	65.4	
	Missing^b^	44	0.6			
Family structure	Parents and grandparent(s)	1439	34.6	2716	67.2	0.770
	Two parents	907	34.0	1757	66.0	
	Single parent	81	32.8	166	65.4	
	Missing	183	2.5			
Father’s employment	Full time	2267	34.3	4345	65.7	0.478
	Part time	13	41.9	18	58.1	
	Not employed	24	39.3	37	60.7	
	Missing	545	7.5			
Mother’s employment	Full time	1281	34.6	2418	65.4	0.944
	Part time	743	34.2	1429	65.8	
	Not employed	351	34.6	663	65.4	
	Missing	364	5.0			
Siblings	Yes	2252	34.2	4337	65.8	0.884
	No	167	34.5	317	65.5	
	Missing	176	2.4			
Frequency of breakfast	Very often	2164	34.5	4106	65.5	0.261
	Often	203	34.7	382	65.3	
	Sometimes	62	30.7	140	69.3	
	Seldom	19	25.3	56	74.7	
	Missing	117	1.6			
Frequency of physical activity	Very often	549	32.5	1142	67.5	0.234
	Often	1071	34.6	2027	65.4	
	Occasionally	702	35.0	1302	65.0	
	Never	115	37.2	194	62.8	
	Missing	147	2.0			
Amount of sleep	<7 hours	500	36.9	855	63.1	0.143
	7–8 hours	1072	34.1	2075	65.9	
	8–9 hours	696	33.1	1405	66.9	
	>9 hours	173	34.2	333	65.8	
	Missing	140	1.9			

SD, standard deviation.

^a^There were 44 participants with missing values for maternal hypertension history.

^b^Proportion of missing values = number of missing values/total sample size (*N* = 7249).

The prevalence of overweight was much higher in children with overweight parents than non-overweight parents (eg 42.5% in children with an overweight mother and 17.0% in children with a mother with a normal BMI; Table 3). The prevalence of overweight was also significantly higher among children with a maternal family history of hypertension than those without (20.9% among children with a maternal family history of hypertension compared with 17.7% among those without). There was no significant difference in the prevalence of overweight between children with a paternal family history of hypertension and those without (19.5% among children with a paternal family history of hypertension compared with 18.1% among those without). When the number of maternal family members with hypertension increased, the prevalence of overweight among children also increased, and this trend was significant; this phenomenon was not observed for paternal family members (Table 3).

**Table 3.** Prevalence of overweight associated with a family history of hypertension and parental overweight

Variable	*n*	Cases	%	χ^2^	*P*
Maternal family history of hypertension^a^					
No	4114	728	17.7	9.293	0.002
Yes	2137	446	20.9		
Missing^c^	998		13.8		
Number of maternal family members with hypertension					
0	4114	728	17.7	18.114	0.000
1	1826	369	20.2		
2	299	71	23.7		
3	12	6	50.0		
Missing	998		13.8		
Paternal family history of hypertension^b^					
No	3976	719	18.1	2.016	0.156
Yes	2215	433	19.5		
Missing	1058		14.6		
Number of paternal family members with hypertension					
0	3976	719	18.1	4.752	0.191
1	1778	336	18.9		
2	396	89	22.5		
3	41	8	19.5		
Missing	1058		14.6		
Maternal overweight					
No	5771	980	17.0	173.141	0.000
Yes	435	185	42.5		
Missing	1044		14.4		
Paternal overweight					
No	4743	767	16.2	104.959	0.000
Yes	1195	348	29.1		
Missing	1312		18.1		

^a^Maternal family members include the mother and the grandfather and grandmother on the mother’s side.

^b^Paternal family members include the father and the grandfather and grandmother on the father’s side.

^c^Proportion of missing values = number of missing values/total sample size (*N* = 7249).

There was no interaction between sex and family history of hypertension on the association between family history of hypertension and childhood overweight (*P* > 0.05). The adjusted OR of overweight for children at age 12 was 1.21 (95% CI 1.04–1.39) for those with a maternal family history of hypertension compared to those without after adjusting for frequency of breakfast, amount of sleep, frequency of physical activity, family structure, and parental overweight (Table 4). The adjusted OR increased from 1.16 (95% CI 0.99–1.35) to 1.42 (95% CI 1.04–1.92) to 4.75 (95% CI 1.35–16.69) as the number of family members with hypertension increased from 1 to 2 to 3, respectively. For overweight male children at age 12, the adjusted OR was 1.25 (95% CI 1.03–1.52) for those with a maternal family history of hypertension compared to those without. The adjusted OR increased from 1.18 (95% CI 0.96–1.45) to 1.58 (95% CI 1.04–2.38) to 5.84 (95% CI 1.28–26.57) among male children when the number of family members with hypertension increased from 1 to 2 to 3, respectively. The relationship between maternal family history of hypertension and childhood overweight was not significant among female children (OR 1.13, 95% CI 0.90–1.41). Further additive scale analysis showed the following: relative excess risk due to interaction = 0.031 (95% CI −1.497–1.558); proportion attributable to interaction = 0.010 (95% CI −0.486–0.506); and synergy index = 1.015 (95% CI 0.482–2.138). These results demonstrate that the interaction between maternal overweight and maternal history of hypertension was not additive.

**Table 4.** Unadjusted and adjusted ORs and 95% CIs for child overweight in relation to parental overweight status and maternal family history of hypertension

Variable	Total				Males				Females			
		
	Unadjusted		Adjusted		Unadjusted		Adjusted		Unadjusted		Adjusted	
					
	OR	95% CI	OR	95% CI	OR	95% CI	OR	95% CI	OR	95% CI	OR	95% CI
Maternal family history of hypertension^a^												
No	1.00		1.00		1.00		1.00		1.00		1.00	
Yes	1.23	1.08–1.39	**1.21**	**1.04–1.39**	1.28	1.07–1.52	1.25	1.03–1.52	1.17	0.96–1.43	1.13	0.90–1.41
Number of maternal family relatives with hypertension^a^												
0	1.00		1.00		1.00		1.00		1.00		1.00	
1	1.18	1.03–1.35	**1.16**	**0.99–1.35**	1.21	1.00–1.52	1.18	0.96–1.45	1.14	0.93–1.41	1.12	0.88–1.41
2	1.45	1.09–1.91	**1.42**	**1.04–1.92**	1.57	1.08–2.28	1.58	1.04–2.38	1.33	0.87–2.02	1.19	0.74–1.91
3	4.65	1.49–14.46	**4.75**	**1.35–16.69**	6.54	1.56–27.47	5.84	1.28–26.57	1.88	0.19–18.09	2.14	0.17–26.49
Maternal overweight^b^												
No	1.00		1.00		1.00		1.00		1.00		1.00	
Yes	3.67	2.99–4.49	3.12	2.49–3.91	3.26	2.44–4.35	2.62	1.89–3.62	4.18	3.15–5.56	3.78	2.75–5.19
Paternal overweight^b^												
No	1.00		1.00		1.00		1.00		1.00		1.00	
Yes	2.15	1.85–2.48	2.11	1.79–2.47	2.41	1.97–2.93	2.31	1.86–2.86	1.89	1.51–2.35	1.91	1.50–2.42

OR, odds ratio; CI, confidence interval.

^a^Odds ratios are adjusted for child overweight at age 3, lifestyle factors (frequency of breakfast, frequency of physical activity, amount of sleep), family structure, and parental overweight status.

^b^Odds ratios are adjusted for child overweight at age 3, lifestyle factors (frequency of breakfast, frequency of physical activity, amount of sleep), family structure, and maternal family history of hypertension.

## DISCUSSION

In the present study, we explored the effect of a family history of hypertension on increases in prevalence of overweight. We found that a maternal family history of hypertension was associated with a greater risk of overweight in children at age 12 and that this association was independent of the family history of obesity.

Family history and personal genomics may well become fundamental infrastructure tools for health and health care this century.^16^ Although most chronic conditions do not manifest until adulthood, an increasing number of studies have indicated that children and adolescents with family histories of certain conditions already show preclinical signs of those conditions. A study of Mexican elementary schools showed that healthy prepubescent children with a maternal family history of hypertension had significantly higher insulin levels than children with a paternal family history of hypertension and those without any family history of hypertension,^17^ and the study suggested that hyperinsulinemia could be the primary metabolic alteration in the offspring of hypertensive mothers. Further, studies have shown that subjects who report a family history of hypertension are much more likely to be obese and to have higher concentrations of serum lipids than those without, which supports the idea of a metabolic syndrome in which insulin resistance provides a common pathway for the development of hypertension, diabetes, and obesity.^6^ The results of the present study also suggest that a maternal family history of hypertension indicates a greater risk of overweight in children, which subsequently increases the risk of diabetes, cardiovascular disease, and other chronic diseases. Further, these findings support the hypothesis that a cardiovascular risk phenotype is transmitted on the maternal lineage with a pattern that indicates mitochondrial DNA-mediated inheritance.^17^ Maternal factors such as dietary intake, central adiposity, and placental insufficiency during gestation may contribute significantly to the programming of an offspring disease phenotype. Fetal growth is determined by the interaction between the fetal genome and the environment, which is determined by the maternal environment and maternal and placental physiology. The developmental origins of this disease paradigm and its underlying mechanistic and evolutionary bases have major implications for addressing the increasing burden of metabolic and cardiovascular disease.^18^ Further research is needed to gain new insights into the genetic etiology of obesity and chronic disease risk in children.

Regardless of the etiology of the disease in question, a healthier lifestyle will help modify the risks. The offspring of hypertensive parents are particularly prone to gaining excess weight,^19^ and lower levels of physical activity linked to increased obesity have been demonstrated among family members of individuals with hypertension.^20^ This increased risk of obesity and decreased level of physical activity among family members suggests that modification of diet and activity patterns could be an effective intervention. People are more likely to be motivated to modify their behavior if they have witnessed the burden of disease firsthand; their motivation for changing their behavior will therefore be higher than that among the general population. The observed risk of obesity in children associated with parental factors might involve a parent-child linkage of environmental factors. For a more effective preventive strategy for obesity in both children and adults, a family-based approach to risk assessment and risk reduction should be considered. The validity and utility of using family history as a screening tool can be largely improved by combining family history with BMI or other measures of adiposity, which can help experts identify high-risk groups and develop targeted intervention programs.

Research on risk factors for child obesity has found that obesity at age 17 is already related to body habitus at birth in girls, whereas among boys it is related to habitus at age 3.^21^ Our study confirms that overweight at age 3 is an independent risk factor for overweight at age 12.^22^ Thus, prevention of obesity should start as early as possible. Prevention of obesity in childhood can help reduce the risk of diabetes mellitus, hypertension, stroke, coronary heart disease, and other chronic diseases in both children and adults.

Although the additive synergistic effect of family history and parental overweight was not statistically significant, the mechanisms for possible synergistic influence of family history of hypertension or other chronic disease and overweight on child overweight need to be explored further.

This study has several strengths. Since parents answered the questionnaire on the presence of chronic disease in both themselves and grandparents, the data were more accurate than they would have been had this information been reported by the child. Despite a lack of consensus on which family history elements contribute to familial risk assessment, self-reported family histories continue to be incorporated into a number of risk assessment tools for common chronic diseases.^3^ Measurements of parents’ height and weight were collected and used to calculate BMI to increase accuracy and reduce recall bias. Finally, we collected data on possible confounders, such as diet, physical activity, and amount of sleep to account for the effects of these confounders.

However, despite these strengths, there are several limitations to our study. First, the diagnosis of hypertension was self-reported, which likely resulted in an underestimation of the actual prevalence of the disease. Hypertension often remains subclinical as long as damage to target organs remains within acceptable limits for an affected individual.^6^ Further, information on family histories may have changed over the lifetimes of the children and their relatives. We collected such information only in Phase 1, when the parents were relatively young, which may have underestimated the actual prevalence of hypertension in the target population. Second, in most cases, the questionnaire was answered by mothers of children, so the paternal family history of diseases and paternal anthropometric data may be underreported and less accurate than maternal data, which may underestimate the risk of paternal family history of hypertension and paternal obesity on child overweight. In addition, maternal history of hypertension and BMI as estimated by self-reported heights and weights may also be underestimated, subsequently leading to an underestimation of the associations of maternal factors with child overweight. In both cases, the true associations of parental factors with child overweight may be much stronger than the estimated associations in this study. Third, the present study is a follow-up study and may have been subject to potential follow-up bias. However, we followed several validated quality control measures during the follow-up in order to minimize these biases.

In conclusion, using longitudinal data from the Toyama Birth Cohort Study, we found that a maternal family history of hypertension is positively associated with the risk of overweight at age 12, with risk increasing as the number of family members (parents or grandparents) with hypertension increases.

## ONLINE ONLY MATERIALS

Abstract in Japanese.
