# Supplementary material for: Family History of Hypertension and the Risk of Overweight in Japanese Children: Results From the Toyama Birth Cohort Study
Source: J Epidemiol. 2014 Jul 5;24(4):304–11. doi: 10.2188/jea.JE20130149 (PMC4074635; doi:10.2188/jea.JE20130149)
Supplement: Abstract in Japanese. [file je-24-304-s001.pdf]

高血圧の家族歴と子どもの過体重リスク：富山出生コホート研究の結果より

Jufen Liu[1,2]、関根道和[2]、立瀬剛志[2]、濱西島子[2]、藤村裕子[2]、Xiaoying Zheng[3]

１）北京大学公衆衛生大学院疫学生物統計学講座
２）富山大学大学院医学薬学研究部疫学健康政策学講座
３）北京大学人口学研究所

【背景】家族歴は、生活習慣病のリスク評価や管理に有用な情報である。しかし、高血圧の家族歴の子どもの過体重への影響については、まだ報告がない。
【方法】富山出生コホート研究の対象者のうち、2002 年の調査まで追跡できた 7249 人（平均 12.3 歳）を対象とした。高血圧の家族歴に関する情報は、子どもの両親や祖父母に高血圧と診断された人がいるかどうかを、子どもの保護者から得た。子どもの過体重の有無は、年齢性別ごとの体格指数を用いた国際分類方法で判断した。
【結果】12 歳の時の過体重の有病率は男子で 21.7%、女子で 15.7%であった。家族構成、両親の雇用状況、生活習慣要因を調整した後、母方に高血圧の既往がある場合の 12 歳の時の子どもの過体重に対するオッズ比は 1.21（95%信頼区間 : 1.04‐1.39）であった。母方の高血圧者の数の 12 歳の時の子どもの過体重に対するオッズ比は、高血圧者が 1 人の場合で 1.16（0.99‐1.35）、高血圧者が 2 人の場合で 1.42（1.04‐1.92）、高血圧者が 3 人の場合で 4.75（1.35‐16.69）であり、母方の高血圧者の数が増えるほど子どもの過体重に対するオッズ比は上昇した。父方の高血圧の家族歴と子どもの過体重との関係は有意ではなかった。
【結論】母方の高血圧の家族歴は、12 歳の時における子どもの過体重のリスクとなる。
